# Effects of catechins, resveratrol, silymarin components and some of their conjugates on xanthine oxidase‐catalyzed xanthine and 6‐mercaptopurine oxidation

**DOI:** 10.1002/jsfa.14045

**Published:** 2024-11-28

**Authors:** Tímea Bencsik, Orsolya Balázs, Róbert G Vida, Balázs Z Zsidó, Csaba Hetényi, Kateřina Valentová, Miklós Poór

**Affiliations:** ^1^ Department of Pharmacognosy, Faculty of Pharmacy University of Pécs Pécs Hungary; ^2^ Department of Pharmaceutics and Central Clinical Pharmacy, Faculty of Pharmacy University of Pécs Pécs Hungary; ^3^ Pharmacoinformatics Unit, Department of Pharmacology and Pharmacotherapy, Medical School University of Pécs Pécs Hungary; ^4^ National Laboratory for Drug Research and Development Budapest Hungary; ^5^ Institute of Microbiology of the Czech Academy of Sciences Prague Czech Republic; ^6^ Department of Laboratory Medicine, Medical School University of Pécs Pécs Hungary; ^7^ Molecular Medicine Research Group, János Szentágothai Research Centre University of Pécs Pécs Hungary

**Keywords:** catechins, resveratrol, silymarin, sulfate conjugates, xanthine oxidase, enzyme inhibition

## Abstract

**BACKGROUND:**

Over the past two decades, the global incidence of gout has markedly increased, affecting people worldwide. Considering the side effects of xanthine oxidase (XO) inhibitor drugs (e.g. allopurinol and febuxostat) used in the treatment of hyperuricemia and gout, the potential application of phytochemicals has been widely studied. In addition, XO also takes part in the elimination of certain drugs, including 6‐mercaptopurine. In the current explorative study, we aimed to examine the potential effects of tea catechins, resveratrol, silymarin flavonolignans and some of their conjugated metabolites on XO‐catalyzed xanthine and 6‐mercaptopurine oxidation, applying *in vitro* assays and modeling studies.

**RESULTS:**

Catechins, resveratrol and resveratrol conjugates exerted no or only weak inhibitory effects on XO. Silybin A, silybin B and isosilybin A were weak, silychristin was a moderate, while 2,3‐dehydrosilychristin was a potent inhibitor of the enzyme. Sulfate metabolites of silybin A, silybin B and isosilybin A were considerably stronger inhibitors compared to the parent flavonolignans, and the sulfation of 2,3‐dehydrosilychristin slightly increased its inhibitory potency. Silychristin was the sole flavonolignan tested, where sulfate conjugation decreased its inhibitory effect.

**CONCLUSION:**

2,3‐Dehydrosilychristin seems to be a promising candidate for examining its *in vivo* antihyperuricemic effects, because both the parent compound and its sulfate conjugate are highly potent inhibitors of XO. © 2024 The Author(s). *Journal of the Science of Food and Agriculture* published by John Wiley & Sons Ltd on behalf of Society of Chemical Industry.

**ABBREVIATIONS**
APU: allopurinol
CAT: catechin
DSC: 2,3‐dehydrosilychristin
DSCS: 2,3‐dehydrosilychristin‐19‐*O*‐sulfate
ECG: epicatechin gallate
ECT: epicatechin
EGC: epigallocatechin
EGCG: epigallocatechin gallate
ISA: isosilybin A
ISAS: isosilybin A‐20‐*O*‐sulfate
R3G: resveratrol‐3‐glucuronide
R3S: resveratrol‐3‐sulfate
RES: resveratrol
SA: silybin A
SAS: silybin A‐20‐*O*‐sulfate
SB: silybin B
SBS: silybin B‐20‐*O*‐sulfate
SC: silychristin
SCS: silychristin‐19‐*O*‐sulfate
XO: xanthine oxidase
6MP: 6‐mercaptopurine

## INTRODUCTION

**Figure 1:**
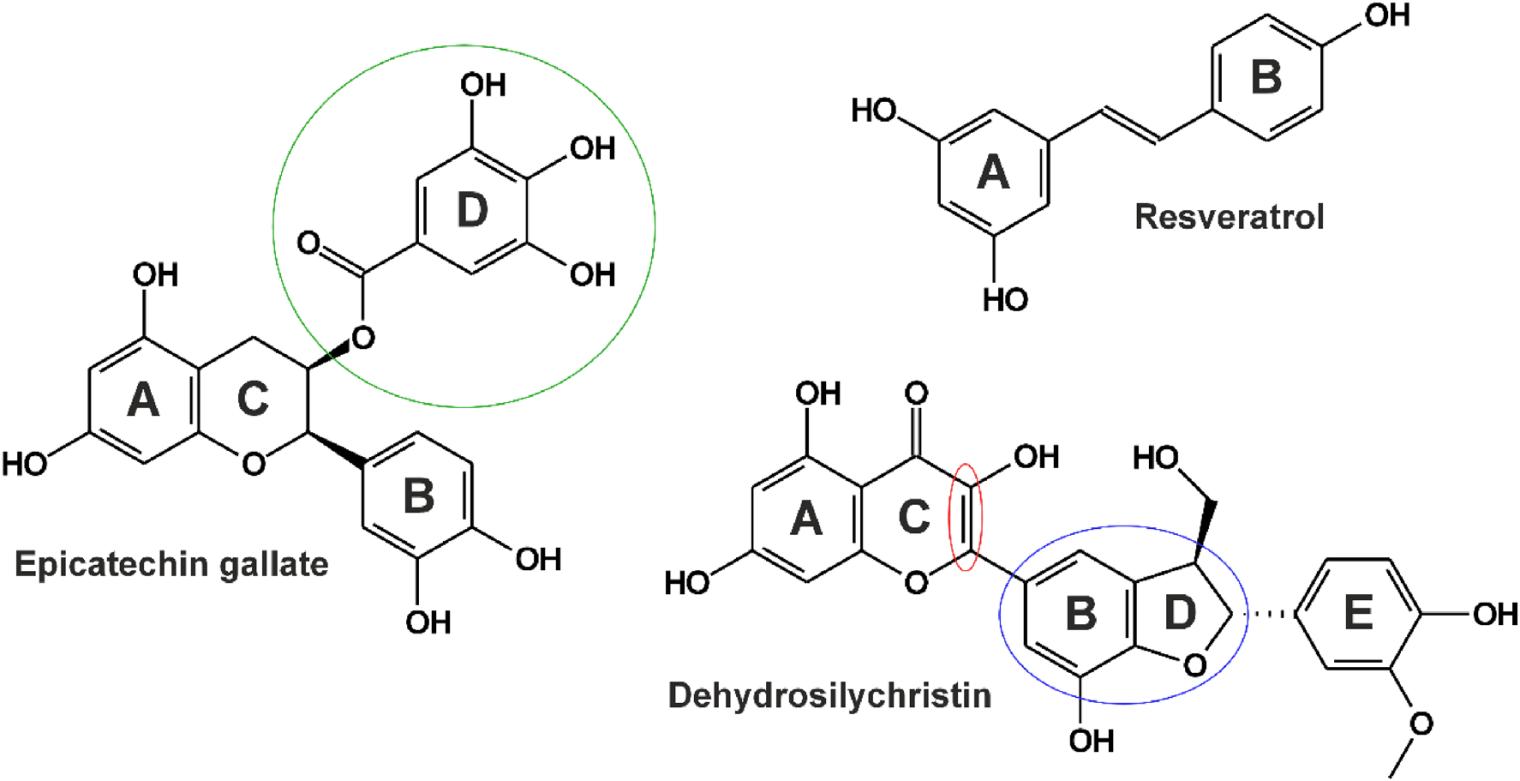
Chemical structures of (−)‐epicatechin gallate (ECG), *trans*‐resveratrol (RES) and 2,3‐dehydrosilychristin (DSC). The gallate ester part of ECG is marked with green. The dihydrobenzofuran structure and the 2–3 double bond in the benzopyran ring of DSC are marked with blue and red, respectively.

Catechins are flavan‐3‐ol derivatives contained in several dietary sources, including tea, coffee, berries, grapes and wine.^1^ The most abundant catechins in tea are catechin (CAT), epicatechin (ECT), epicatechin gallate (ECG; Fig. 1), epigallocatechin (EGC) and epigallocatechin gallate (EGCG).^2^ After the consumption of black or green tea, the total catechin levels in the circulation were typically between 100 and 600 nmol L^−1^.^2^, ^3^ Catechins may have beneficial health effects, e.g. their antioxidant, anti‐inflammatory and anticancer impacts have been suggested.^1^

Resveratrol (RES; Fig. 1) is a natural polyphenol. Structurally it is a member of the stilbenoid group. RES occurs in berries, peanuts, grapes and red wine, for example, and it is also contained in certain dietary supplements.^4^ RES showed promising effects in the treatment of some neurological disorders, cardiovascular diseases and diabetes in clinical trials.^5^ The oral bioavailability of RES is less than 1%, due to its high presystemic biotransformation by sulfotransferases and uridine 5′‐diphosphoglucuronosyltransferases.^6^ Based on human and animal studies, sulfate and glucuronide conjugates of RES appear at high concentrations in the circulation and in certain tissues.^7^, ^8^, ^9^, ^10^ The peroral administration of high doses (0.5–5.0 g) of RES resulted in micromolar (even 10–20 μmol L^−1^) peak plasma concentrations of resveratrol‐3‐sulfate (R3S) and resveratrol‐3‐glucuronide (R3G) in humans.^10^, ^11^

Silymarin is a polyphenolic fraction from the extract of milk thistle (*Silybum marianum* (L.) Gaertner) fruit with widely known hepatoprotective effects.^12^ The major components of silymarin are silybin A (SA) and silybin B (SB); however, other important flavonolignans are also present, including isosilybin A (ISA), silychristin (SC), 2,3‐dehydrosilychristin (DSC; Fig. 1) and silydianin.^13^, ^14^ The main circulating metabolites of silymarin components in humans are glucuronide and sulfate derivatives.^15^, ^16^ After the *per os* administration of milk thistle extract (100–360 mg) to healthy human volunteers, the peak plasma concentrations of the flavonolignans were from the nanomolar to the low micromolar range.^17^, ^18^

Gout is a chronic disease caused by the imbalance of urate homeostasis. Elevated uric acid concentrations in the joints can result in the precipitation of urate crystals, leading to sterile inflammation, intensive pain of the joints and their limited range of motion.^19^ In addition, hyperuricemia and gout can be associated with numerous other disorders, including hypertension, myocardial infarction, stroke, obesity, hyperlipidemia, type 2 diabetes mellitus and chronic kidney disease.^20^ Gout has a large global incidence: in the last 30 years, the number of people suffer from this disease has increased from 22 to 53 million.^19^ The chronic pharmacotherapy of gout is based on three major strategies: inhibition of uric acid production (e.g. xanthine oxidase (XO) inhibitors), enhancement of the renal excretion of uric acid (e.g. uricosurics) and the degradation of uric acid produced (e.g. application of recombinant uricases).^20^ The potent XO inhibitor drugs (e.g. allopurinol (APU) and febuxostat) have high importance in the chronic treatment of gout; nowadays, APU is the first‐line urate‐lowering agent applied.^20^, ^21^

Xanthine oxidoreductase is a molybdoprotein with two iron–sulfur centers (Fe_2_S_2_) and flavin adenine dinucleotide (FAD) co‐factors; its two interconvertible forms are XO and xanthine dehydrogenase.^22^ Both forms catalyze the oxidation of hypoxanthine to xanthine then of xanthine to uric acid (the end product of purine catabolism in humans). XO can only reduce oxygen, while xanthine dehydrogenase also reduces NAD^+^ (more preferably compared to oxygen).^23^ In the presence of abundant NAD^+^ supply, the xanthine dehydrogenase‐catalyzed formation of superoxide anion radical is limited because NAD^+^ is the favored electron acceptor of this form.^23^ However, during the reoxidation of fully reduced XO, it generates superoxide anion radicals and hydrogen peroxide, also leading to the higher production of hydroxyl radical and peroxynitrite.^22^ Based on earlier reports, a more than threefold variation in xanthine oxidoreductase activity has been observed in humans.^23^ Due to the XO‐mediated radical formation, the enzyme can take part in the pathogenesis of several (e.g. cardiovascular) diseases;^20^ thus, the inhibition of XO may have further beneficial health effects. However, due to the controversial results, further *in vivo* studies and clinical trials are required to prove this hypothesis.^22^, ^23^, ^24^

Based on *in vitro* experiments, certain natural polyphenols proved to be potent inhibitors of XO;^25^ however, the typically low oral bioavailability of these compounds limits their *in vivo* antihyperuricemic effects.^26^ Importantly, some conjugated metabolites of flavonoids showed similar or even stronger inhibitory actions on XO compared to their parent aglycones.^27^, ^28^ Considering the significant presystemic biotransformation of polyphenols, the potential *in vivo* impact of these derivatives may have high pharmacological importance.

Earlier reports suggest that gallate esters (e.g. ECG, EGCG, and gallocatechin gallate) bind with higher affinity to XO than CAT, ECT, EGC or gallocatechin;^29^ therefore, they are stronger inhibitors of the enzyme.^30^, ^31^ Other studies demonstrated the weak inhibitory impact of RES on XO;^32^, ^33^ however, the potential interactions of its major conjugates (R3S and R3G) have not been examined yet. Furthermore, silybin also proved to be a weak inhibitor of XO,^34^, ^35^ while no data are available regarding other silymarin components and their sulfate derivatives.

XO‐catalyzed biotransformation also plays an important role in the elimination of certain drugs, including 6‐mercaptopurine (6MP) applied for the treatment of cancer and certain autoimmune diseases.^36^ XO is responsible for the oxidation of 6MP to 6‐thiouric acid. The inhibition of this process (e.g. by APU) can compromise the elimination of 6MP leading to the development of severe side effects (e.g. myelosuppression).^37^ Therefore, the application of XO inhibitors (when a patient also suffers from gout) during the administration of 6MP must be carefully considered.

In previous studies, the inhibitory actions of catechins, RES and silybin were tested on XO‐catalyzed xanthine oxidation,^29^, ^30^, ^31^, ^32^, ^33^, ^34^, ^35^ while the effects of these compounds on 6MP oxidation have not been examined yet. It may provide interesting data because some polyphenols showed substrate‐specific inhibition of XO. For example, pyrogallol was a more potent inhibitor of xanthine oxidation, while 3‐phenylpropionic acid proved to be a stronger inhibitor of 6MP oxidation.^27^ In addition, even the positive control inhibitor APU showed major differences in its inhibitory potency with these two substrates.^27^, ^28^ Therefore, we felt it reasonable to compare the impacts of polyphenols on XO‐mediated xanthine *versus* 6MP oxidations.

The two major goals of our *in vitro* explorative study were to identify novel, highly potent inhibitors of XO, and to get an insight into the potential involvement of conjugated metabolites regarding polyphenol–XO interactions. Therefore, the inhibitory effects of CAT, ECT, ECG, EGC, EGCG, RES, R3S, R3G, SA, silybin A‐20‐*O*‐sulfate (SAS), SB, silybin B‐20‐*O*‐sulfate (SBS), ISA, isosilybin A‐20‐*O*‐sulfate (ISAS), SC, silychristin‐19‐*O*‐sulfate (SCS), DSC and 2,3‐dehydrosilychristin‐19‐*O*‐sulfate (DSCS) were examined (see their chemical structures in supporting information, Fig. S1) on XO‐catalyzed xanthine and 6MP oxidation. Since certain silymarin components/metabolites proved to be highly potent inhibitors of the enzyme, their interactions with XO were also investigated using fluorescence quenching experiments. Finally, molecular modeling studies were performed for a deeper understanding of the interactions of SB, SBS, DSC and DSCS with XO.

## MATERIALS AND METHODS

### Reagents

RES, XO (from bovine milk), xanthine, uric acid, APU and 6MP were purchased from Merck (Darmstadt, Germany). SA, SB, ISA, SC, DSC, SAS, SBS, ISAS, SCS and DSCS were isolated or synthesized as earlier reported.^38^, ^39^ CAT and ECT were purchased from Extrasynthese (Genay, France). ECG, EGC, EGCG, R3G and 6‐thiouric acid were obtained from Biosynth (Berkshire, UK). R3S was from Toronto Research Chemicals (Toronto, Canada).

Stock solutions of polyphenols (10 mmol L^−1^), xanthine (2 mmol L^−1^), 6MP (2 mmol L^−1^), 6‐thiouric acid (2 mmol L^−1^) and APU (5 mmol L^−1^) were prepared in dimethyl sulfoxide (DMSO; spectroscopic grade; Fluka, Charlotte, NC, USA) and stored at −20 °C. Uric acid (2 mmol L^−1^) was dissolved in 0.01 mol L^−1^ sodium hydroxide.

### XO assay with xanthine as substrate

The effects of polyphenols on XO‐catalyzed xanthine oxidation were tested using our previously reported method, without modification.^28^ Briefly, in a 500 μL final volume, xanthine (5 μmol L^−1^), XO (0.0003 U mL^−1^) and polyphenols (0–50 μmol L^−1^) were incubated in a thermomixer for 8 min at 700 rpm and 37 °C, in sodium phosphate buffer (0.05 mol L^−1^, pH 7.5). The reaction was started with the enzyme and stopped with 30 μL of perchloric acid solution (6 mol L^−1^). After vortexing, 195 μL of potassium hydroxide solution (1 mol L^−1^) was added, then samples were cooled to 4 °C and centrifuged for 5 min at 14 000 × *g* and 4 °C. The supernatant was directly analyzed using high‐performance liquid chromatography (HPLC) with UV detection (see details in a subsequent subsection). We employed solvent controls (DMSO) in all experiments and used APU as positive control inhibitor. Even in the presence of the highest level (50 μmol L^−1^) of polyphenols applied, DMSO (0.5% v/v) did not affect XO‐catalyzed xanthine oxidation.

To examine the reversibility of the SBS‐, DSC‐ and DSCS‐induced inhibitory actions on XO, the following experiment was carried out.^40^ Standard levels of XO (final concentration: 0.0003 U mL^−1^) and polyphenols (final concentrations: 5 μmol L^−1^ SBS and 0.5 μmol L^−1^ DSC and DSCS) were preincubated for 10 min (700 rpm, 37 °C), then the reaction was started with the addition of increasing amounts of xanthine (final concentrations: 5, 25 and 50 μmol L^−1^). After 8 min incubation (700 rpm, 37 °C), the reaction was stopped, and the samples were treated in the same way as described in the previous paragraph.

### XO assay with 6MP as substrate

The effects of polyphenols on XO‐catalyzed 6MP oxidation were tested using our previously reported method, without modification.^28^ Briefly, in a 500 μL final volume, 6MP (5 μmol L^−1^), XO (0.01 U mL^−1^) and polyphenols (0–50 μmol L^−1^) were incubated in a thermomixer for 25 min at 700 rpm and 37 °C, in sodium phosphate buffer (0.05 mol L^−1^, pH 7.5). Other experimental details were the same as described in the previous subsection. We used solvent controls (DMSO) in each experiment and employed APU as positive control inhibitor. Even in the presence of the highest level (50 μmol L^−1^) of polyphenols applied, DMSO (0.5% v/v) did not influence XO‐catalyzed 6MP oxidation.

### HPLC analyses

For the analysis of xanthine and uric acid, a Dionex Ultimate 3000 HPLC system (Thermo Scientific; Waltham, MA, USA) was used, containing a pump (LPG‐3400 SD), an autosampler column compartment (ACC‐3000; with integrated autosampler and column oven), a diode‐array detector (DAD‐3000) and Dionex Chromeleon 7 software. We applied our previously reported HPLC‐UV method.^40^ Briefly, samples of 20 μL injected volume were driven through a precolumn (C18, 4.0 × 3.0 mm, Security Guard Cartridge; Phenomenex, Torrance, CA, USA) linked to a Kinetex EVO C18 analytical column (250 × 4.6 mm, 5 μm; Phenomenex). The isocratic elution was carried out applying sodium phosphate buffer (10 mmol L^−1^, pH 4.55) and methanol (98:2% v/v) as mobile phase (flow rate: 1.0 mL min^−1^) at room temperature. Peak areas were evaluated at 275 nm (see the representative chromatogram in supporting information, Fig. S2). The major validation parameters of this HPLC assay were the following: linearity (0.2–5.0 μmol L^−1^), *R* ^2^ = 0.999 for xanthine and *R* ^2^ = 0.999 for uric acid; limit of detection (signal‐to‐noise ratio of 3), 0.03 μmol L^−1^ for xanthine and 0.04 μmol L^−1^ for uric acid; limit of quantification (signal‐to‐noise ratio of 10), 0.09 μmol L^−1^ for xanthine and 0.12 μmol L^−1^ for uric acid; intraday precision (*n* = 7), 2.5% for xanthine and 3.4% for uric acid.

For the analysis of 6MP and 6‐thiouric acid, an integrated HPLC system (Jasco, Tokyo, Japan) was used, including a binary pump (PU‐4180), an autosampler (AS‐4050), a UV detector (UV‐470) and ChromNAV2 software. Our previously reported HPLC‐UV method was applied.^27^ Briefly, samples of 20 μL injected volume were driven through a precolumn (C18, 4.0 × 3.0 mm, Security Guard Cartridge; Phenomenex) linked to a Gemini NX‐C18 analytical column (C18, 150 × 4.6 mm, 3 μm; Phenomenex). The isocratic elution was carried out applying 0.02 mol L^−1^ phosphoric acid solution, acetonitrile and methanol (91:5:4% v/v) as mobile phase (flow rate: 0.8 mL min^−1^) at room temperature. Peak areas were evaluated at 334 nm (see the representative chromatogram in supporting information, Fig. S2). The major validation parameters of this HPLC assay were the following: linearity (0.2–5.0 μmol L^−1^), *R* ^2^ = 0.999 for 6MP and *R* ^2^ = 0.998 for 6‐thiouric acid; limit of detection (signal‐to‐noise ratio of 3), 0.03 μmol L^−1^ for 6MP and 0.02 μmol L^−1^ for 6‐thiouric acid; limit of quantification (signal‐to‐noise ratio of 10), 0.09 μmol L^−1^ for 6MP and 0.06 μmol L^−1^ for 6‐thiouric acid; intraday precision (*n* = 7), 1.2% for 6MP and 2.0% for 6‐thiouric acid.

### Fluorescence quenching studies

Increasing concentrations of polyphenols (0, 2, 4, 6, 8 and 10 μmol L^−1^) were added to a standard amount of XO (0.4 μmol L^−1^) in sodium phosphate buffer (0.05 mol L^−1^, pH 7.5). Then, the emission spectra of XO were recorded using 280 nm excitation wavelength. The inner‐filter effects of silymarin components/metabolites were corrected based on the following equation^39^, ^41^, ^42^:

(1) $$I_{\mathrm{cor}}=I_{\mathrm{obs}}\;\times \;e^{\left(A_{\mathrm{ex}}+A_{\mathrm{em}}\right)/2}$$

where *I*_cor_ is the corrected and *I*_obs_ is the observed emission intensity; while *A*_ex_ and *A*_em_ are the absorbance of polyphenols at the excitation and emission wavelengths applied, respectively. Polyphenol‐induced fluorescence quenching was evaluated at 335 nm. Stern–Volmer quenching constants (*K*_SV_; unit: L mol^−1^) of polyphenol–XO complexes were determined using the graphical application of the Stern–Volmer equation^43^, ^44^, ^45^:

(2) $$\frac{I_{0}}{I}=1+K_{\mathrm{SV}}\times \left[Q\right]$$

where *I*_0_ and *I* are the emission signals of XO without and with the polyphenols tested, respectively, and [Q] denotes the molar concentration (mol L^−1^) of the quencher (polyphenols). The binding constants (*K*; unit: L mol^−1^) of polyphenol–XO complexes were calculated applying the modified Stern–Volmer equation^43^, ^44^, ^45^:

(3) $$\frac{I_{0}}{\left(I_{0}-I\right)}=\frac{1}{f_{a}\;\times \;K}\times \frac{1}{\left[Q\right]}+\frac{1}{f_{a}}$$

where *I*_0_, *I* and [Q] have the same meaning as in Eqn (2), and *f*_a_ is the fraction of the accessible fluorophore.

### Data analyses

The mean ± standard error of the mean (SEM) values represented are derived from three independent experiments. Statistically significant differences (*P* < 0.05 and *P* < 0.01) were established based on one‐way analysis of variance and Tukey's *post hoc* tests, using SPSS Statistics software (IBM, Armonk, NY, USA).

After we quantified the concentrations of the substrate (*c*_substrate_; xanthine or 6MP) and the product (*c*_product_; uric acid or 6‐thiouric acid) in the incubates, the metabolite formation rate (*R*) was calculated:

(4) $$R\;\left(\%\right)=100\;\times \;\frac{c_{\text{product}}}{\left(c_{\text{substrate}}+c_{\text{product}}\right)}$$

Thereafter, these *R* values were compared in the absence (*R*_control_) and in the presence (*R*_inhibitor_) of inhibitors:

(5) $$\text{Metabolite formation}\;\left(\%\right)=100\;\times \;\frac{R_{\text{inhibitor}}}{R_{\text{control}}}$$

**Figure 2:**
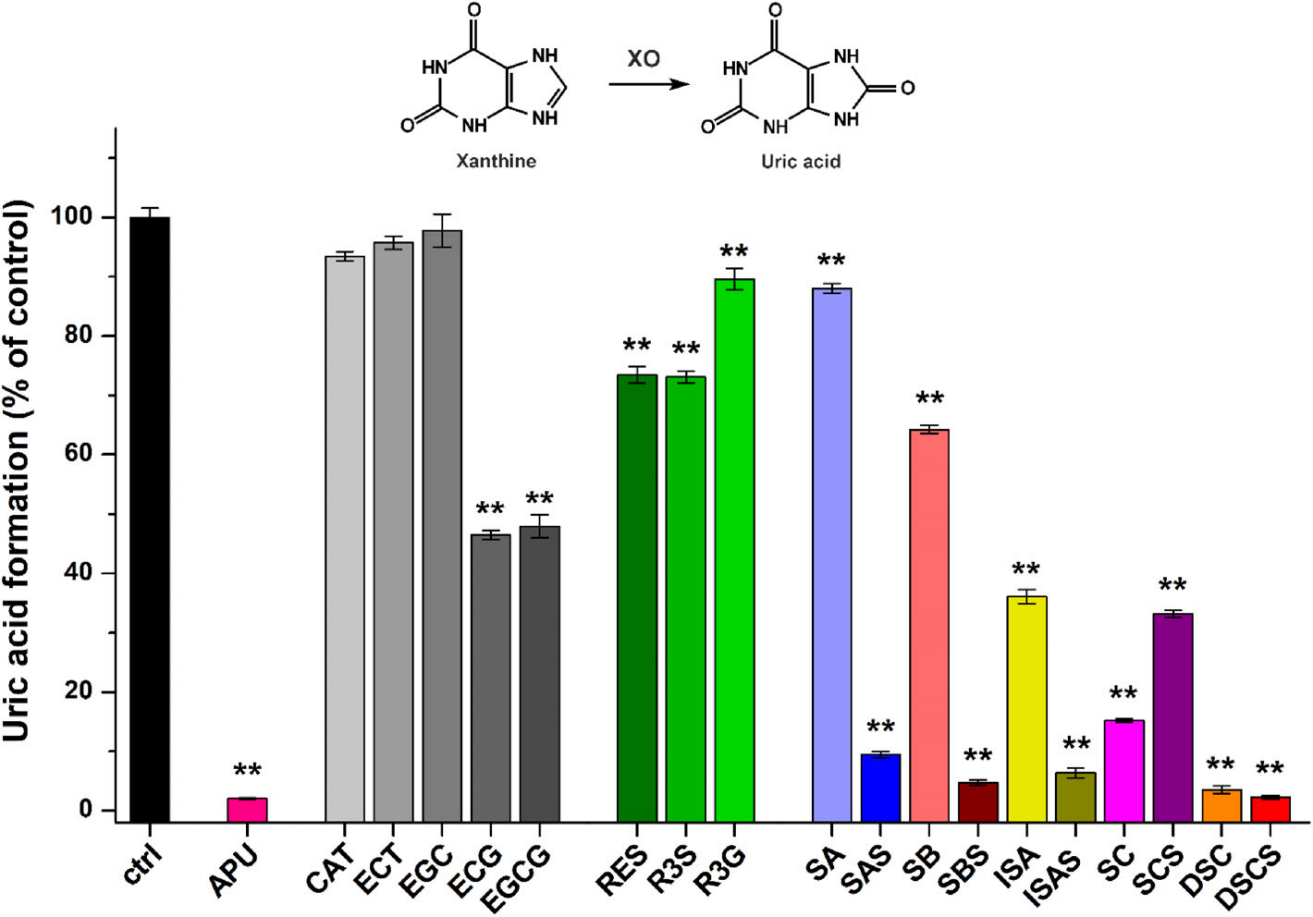
Effects of polyphenols on XO‐catalyzed xanthine oxidation (XO: 0.0003 U mL^−1^; substrate: 5 μmol L^−1^; polyphenols: 20 μmol L^−1^; incubation: 8 min, 700 rpm, 37 °C; *n* = 3; **P < 0.01; CAT, catechin; ECT, epicatechin; ECG, epicatechin gallate; EGC, epigallocatechin; EGCG, epigallocatechin gallate; RES, resveratrol; R3S, resveratrol‐3‐*O*‐sulfate; R3G, resveratrol‐3‐*O*‐*β*‐d‐glucuronide; SA, silybin A; SAS, silybin A‐20‐*O*‐sulfate; SB, silybin B; SBS, silybin B‐20‐*O*‐sulfate; ISA, isosilybin A; ISAS, isosilybin A‐20‐*O*‐sulfate; SC, silychristin; SCS, silychristin‐19‐*O*‐sulfate; DSC, 2,3‐dehydrosilychristin; DSCS, 2,3‐dehydrosilychristin‐19‐*O*‐sulfate).

**Figure 3:**
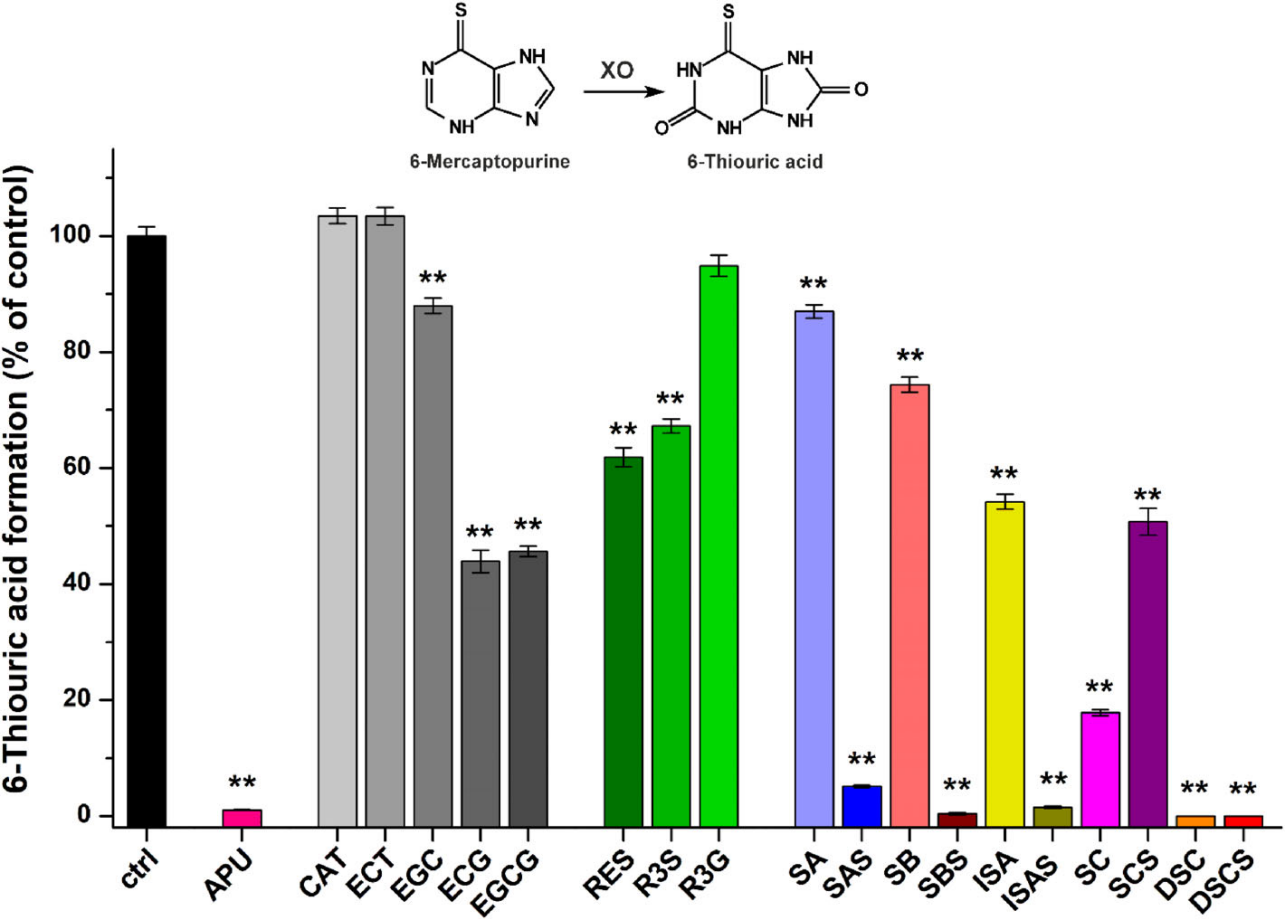
Effects of polyphenols on XO‐catalyzed 6MP oxidation (XO: 0.01 U mL^−1^; substrate: 5 μmol L^−1^; polyphenols: 20 μmol L^−1^; incubation: 25 min, 700 rpm, 37 °C; *n* = 3; **P < 0.01; CAT, catechin; ECT, epicatechin; ECG, epicatechin gallate; EGC, epigallocatechin; EGCG, epigallocatechin gallate; RES, resveratrol; R3S, resveratrol‐3‐*O*‐sulfate; R3G, *trans*‐resveratrol‐3‐*O*‐β‐d‐glucuronide; SA, silybin A; SAS, silybin A‐20‐*O*‐sulfate; SB, silybin B; SBS, silybin B‐20‐*O*‐sulfate; ISA, isosilybin A; ISAS, isosilybin A‐20‐*O*‐sulfate; SC, silychristin; SCS, silychristin‐19‐*O*‐sulfate; DSC, 2,3‐dehydrosilychristin; DSCS, 2,3‐dehydrosilychristin‐19‐*O*‐sulfate).

**Figure 4:**
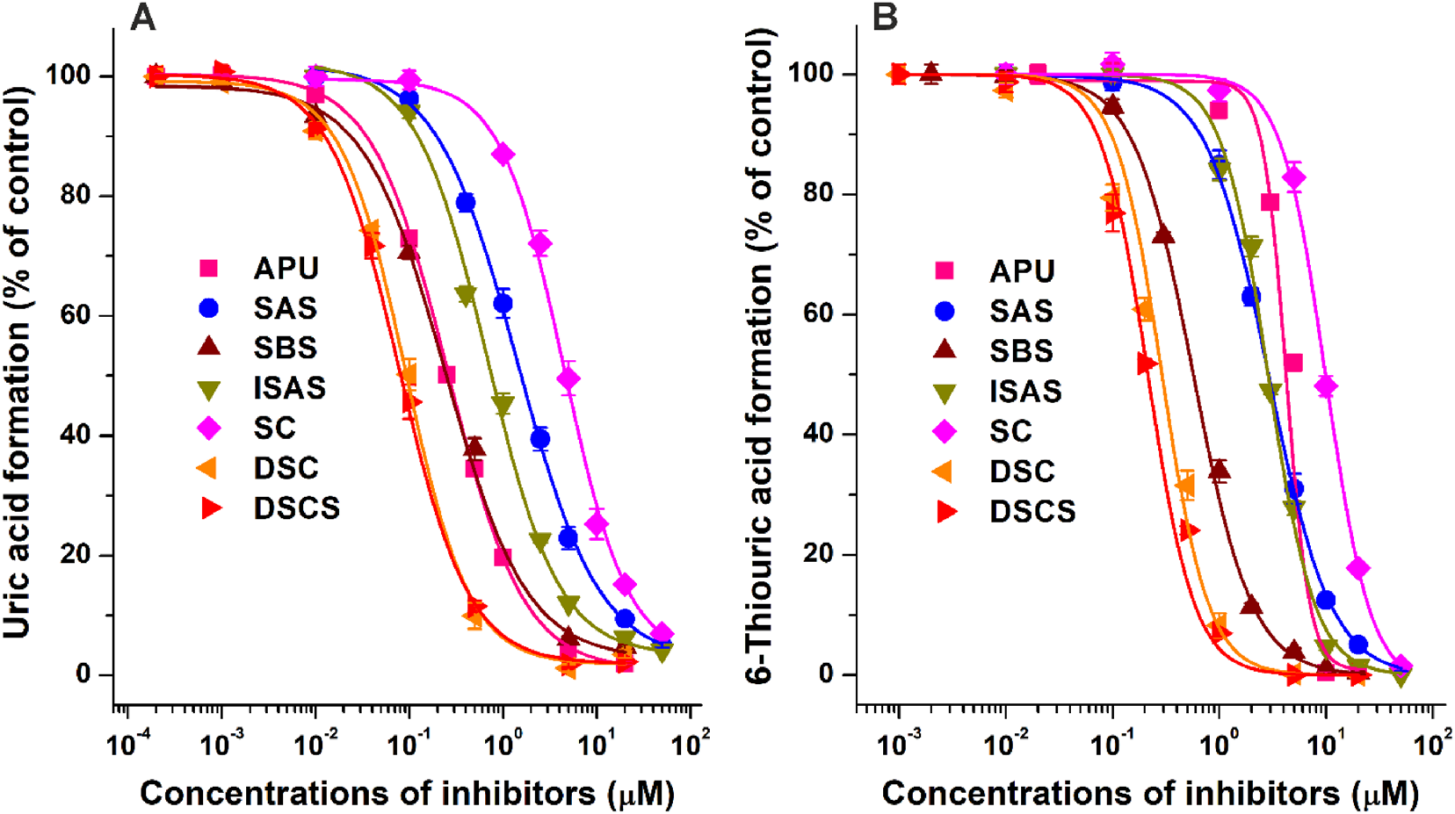
Concentration‐dependent inhibition of XO‐catalyzed xanthine (A) and 6MP (B) oxidation by allopurinol (APU, positive control inhibitor), silybin A‐20‐*O*‐sulfate (SAS), silybin B‐20‐*O*‐sulfate (SBS), isosilybin A‐20‐*O*‐sulfate (ISAS), silychristin (SC), 2,3‐dehydrosilychristin (DSC) and 2,3‐dehydrosilychristin‐19‐*O*‐sulfate (DSCS) (substrate: 5 μmol L^−1^; polyphenols: 0–50 μmol L^−1^; *n* = 3).

These metabolite formation (%) data are presented in Figs 2, 3, 4. To determine IC_50_ values, sigmoidal fitting (Hill1) was performed with Origin software (OriginLab Corporation, Northampton, MA, USA).

### Modeling studies

The structures of SB ((2*R*,3*R*)‐3,5,7‐trihydroxy‐2‐[(2*S*,3*S*)‐3‐(4‐hydroxy‐3‐methoxyphenyl)‐2‐(hydroxymethyl)‐2,3‐dihydro‐1,4‐benzodioxin‐6‐yl]‐2,3‐dihydrochromen‐4‐one), SBS, DSC (3,5,7‐trihydroxy‐2‐[(2*R*,3*S*)‐7‐hydroxy‐2‐(4‐hydroxy‐3‐methoxyphenyl)‐3‐(hydroxymethyl)‐2,3‐dihydro‐1‐benzofuran‐5‐yl]chromen‐4‐one) and DSCS were built in Maestro (Schrödinger Release 2024‐1: Maestro, Schrödinger; New York, NY, USA).^40^, ^46^, ^47^ A steepest descent local energy minimization was performed in Maestro with default settings to ensure a favorable geometry for docking.

Atomic coordinates of XO were obtained from the Protein Data Bank (PDB) with PDB code 3eub,^48^ according to our previous studies.^27^, ^40^ The target was prepared exactly as in our earlier report.^40^

Ligands were docked focused on the active center of XO using AutoDock 4.2.6,^49^ where the docking box was centered on the molybdenum cofactor (MoCo). Lamarckian genetic algorithm was used, 100 docking runs were performed for each ligand and the resulting ligand conformations were clustered and ranked by their free energy of binding (Δ*G*_binding_).^50^ A lower rank indicates a more favorable calculated Δ*G*_binding_ value. Representative docked ligand conformations with the best Δ*G*_binding_ were used for subsequent evaluations.^51^ Re‐docking of xanthine to XO was performed in our earlier study for the validation of the docking method, achieving an excellent match with the experimental ligand binding mode (root mean squared deviation of 1 Å).^27^

## RESULTS

### Inhibitory effects of flavonoids on XO‐catalyzed xanthine and 6MP oxidation

In the first experiment, the impacts of polyphenols were tested on XO‐catalyzed xanthine oxidation at 20 μmol L^−1^ concentration (fourfold greater compared to the substrate). The positive control inhibitor APU almost completely abolished metabolite formation (Fig. 2). CAT, ECT and EGC did not cause significant changes in the activity of the enzyme; nevertheless, ECG and EGCG induced approximately 50% decrease in uric acid production. RES and R3S showed similar inhibitory effects, resulting in 27% inhibition, while R3G caused only a 10% decrease. Among the silymarin components, SA and SB were only weak inhibitors; however, ISA, SC and DSC induced 64%, 85% and 96% decline in metabolite formation, respectively. Despite the weak to moderate impacts of SA, SB and ISA, their sulfate derivatives proved to be significantly stronger inhibitors of xanthine oxidation (Fig. 2). DSCS also showed slightly stronger impact than DSC, while SCS was the sole sulfate derivative that produced a weaker effect compared to its parent compound (SC).

In the following experiment, the effects of polyphenols were also examined on XO‐catalyzed 6MP oxidation at 20 μmol L^−1^ concentration (fourfold greater compared to the substrate). We noticed results mostly similar to those in the xanthine assay. Again, ECG, EGCG, RES, R3S, SA, SB, ISA and SCS proved to be weak to moderate inhibitors, while APU (positive control), SAS, SBS, ISAS, SC, DSC and DSCS caused marked decreases in 6‐thiouric acid formation (Fig. 3). In the 6MP assay, EGC showed statistically significant (*P* < 0.01) but only slight inhibitory action; however, the impact of R3G was not significant (*P* < 0.05). As further differences, ISA and SCS caused almost 20% lower decreases in metabolite formation than in the xanthine assay.

### Concentration‐dependent effects of polyphenols and their metabolites on XO

Since SAS, SBS, ISAS, SC, DSC and DSCS caused more than 80% inhibition in both xanthine and 6MP assays, we examined their concentration‐dependent inhibitory actions on XO. DSCS, DSC and SBS showed the most potent inhibitory effects on XO with both substrates (Fig. 4) with nanomolar IC_50_ values (Table 1). In addition, even at 5 μmol L^−1^ concentration, DSCS, DSC and SBS almost completely abolished XO‐catalyzed uric acid and 6‐thiouric acid formation (Fig. 4).

**Table 1:** IC_50_ values of allopurinol (APU, positive control inhibitor), silybin A‐20‐*O*‐sulfate (SAS), silybin B‐20‐*O*‐sulfate (SBS), isosilybin A‐20‐*O*‐sulfate (ISAS), silychristin (SC), 2,3‐dehydrosilychristin (DSC) and 2,3‐dehydrosilychristin‐19‐*O*‐sulfate (DSCS) regarding XO‐catalyzed xanthine and 6MP oxidation

Inhibitor	Xanthine oxidation		6MP oxidation		IC_50(6MP)_/IC_50(xanthine)_
	IC_50_ (μmol L^−1^)	*α* ^a^	IC_50_ (μmol L^−1^)	*α* ^a^	
APU	0.26	1.00	4.22	1.00	16.2
SAS	1.43	5.50	2.91	0.69	2.0
SBS	0.24	0.92	0.57	0.14	2.4
ISAS	0.68	2.62	2.86	0.68	4.2
SC	4.50	17.3	10.0	2.38	2.2
DSC	0.09	0.35	0.29	0.07	3.2
DSCS	0.08	0.31	0.21	0.05	3.0

^a^ *α* = IC_50_ of flavonoid/IC_50_ of positive control.

In the xanthine assay, the effect of SBS (IC_50_ = 0.24 μmol L^−1^) was similarly strong, while DSCS (IC_50_ = 0.08 μmol L^−1^) and DSC (IC_50_ = 0.09 μmol L^−1^) were more potent inhibitors than the positive control APU (IC_50_ = 0.26 μmol L^−1^) (Fig. 4(A)). Furthermore, ISAS (IC_50_ = 0.68 μmol L^−1^) and SAS (IC_50_ = 1.4 μmol L^−1^) can also be considered as strong inhibitors, while SC as a moderate inhibitor (IC_50_ = 4.5 μmol L^−1^).

In the 6MP assay, SAS and ISAS were slightly stronger and SC was a weaker inhibitor compared to APU (IC_50_ = 4.2 μmol L^−1^). However, DSCS (IC_50_ = 0.21 μmol L^−1^), DSC (IC_50_ = 0.29 μmol L^−1^) and SBS (IC_50_ = 0.57 μmol L^−1^) showed approximately 20‐fold, 15‐fold and 7‐fold stronger inhibition than APU, respectively. Interestingly, SAS, SBS, ISAS, SC, DSC and DSCS were two‐ to fourfold stronger inhibitors of xanthine oxidation *versus* 6MP oxidation (Table 1).

### Testing the reversibility of the inhibitory effects of SBS, DSC and DSCS on xanthine oxidation

**Figure 5:**
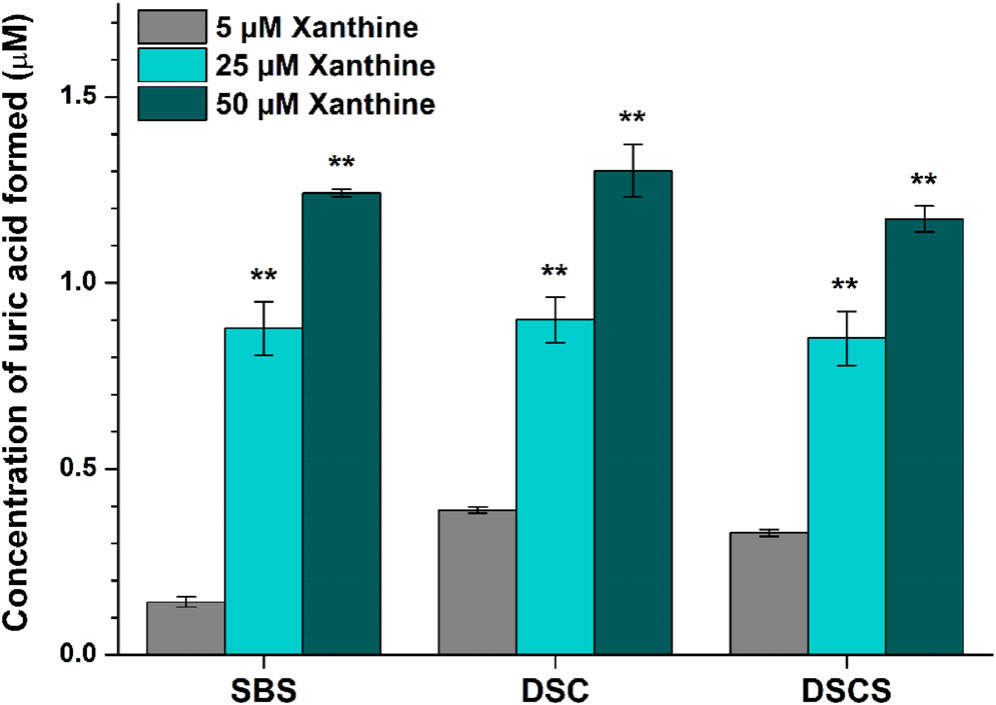
XO‐catalyzed uric acid formation in the presence of silybin B‐20‐*O*‐sulfate (SBS, 5 μmol L^−1^), 2,3‐dehydrosilychristin (DSC, 0.5 μmol L^−1^) or 2,3‐dehydrosilychristin‐19‐*O*‐sulfate (DSCS, 0.5 μmol L^−1^M), with increasing concentrations of xanthine (5, 25 or 50 μmol L^−1^). Polyphenols were preincubated with the enzyme (0.0003 U mL^−1^) for 10 min (700 rpm, 37 °C), then the reaction was started with the addition of xanthine and the samples were incubated for a further 8 min (700 rpm, 37 °C). Uric acid production in the presence of 25 and 50 μmol L^−1^ xanthine was compared to the product formation determined with 5 μmol L^−1^ substrate concentration (**P < 0.01).

To examine the reversibility of SBS‐, DSC‐, and DSCS‐induced inhibition on XO, xanthine assay was also performed with increasing substrate concentrations in the presence of standard levels of the enzyme and the inhibitors. XO was preincubated with the polyphenols and then the reaction was started with the addition of xanthine. In a concentration‐dependent fashion, increasing levels of xanthine significantly elevated uric acid production (Fig. 5), demonstrating that the inhibitory actions of SBS, DSC and DSCS are reversible.

### Fluorescence quenching studies

**Figure 6:**
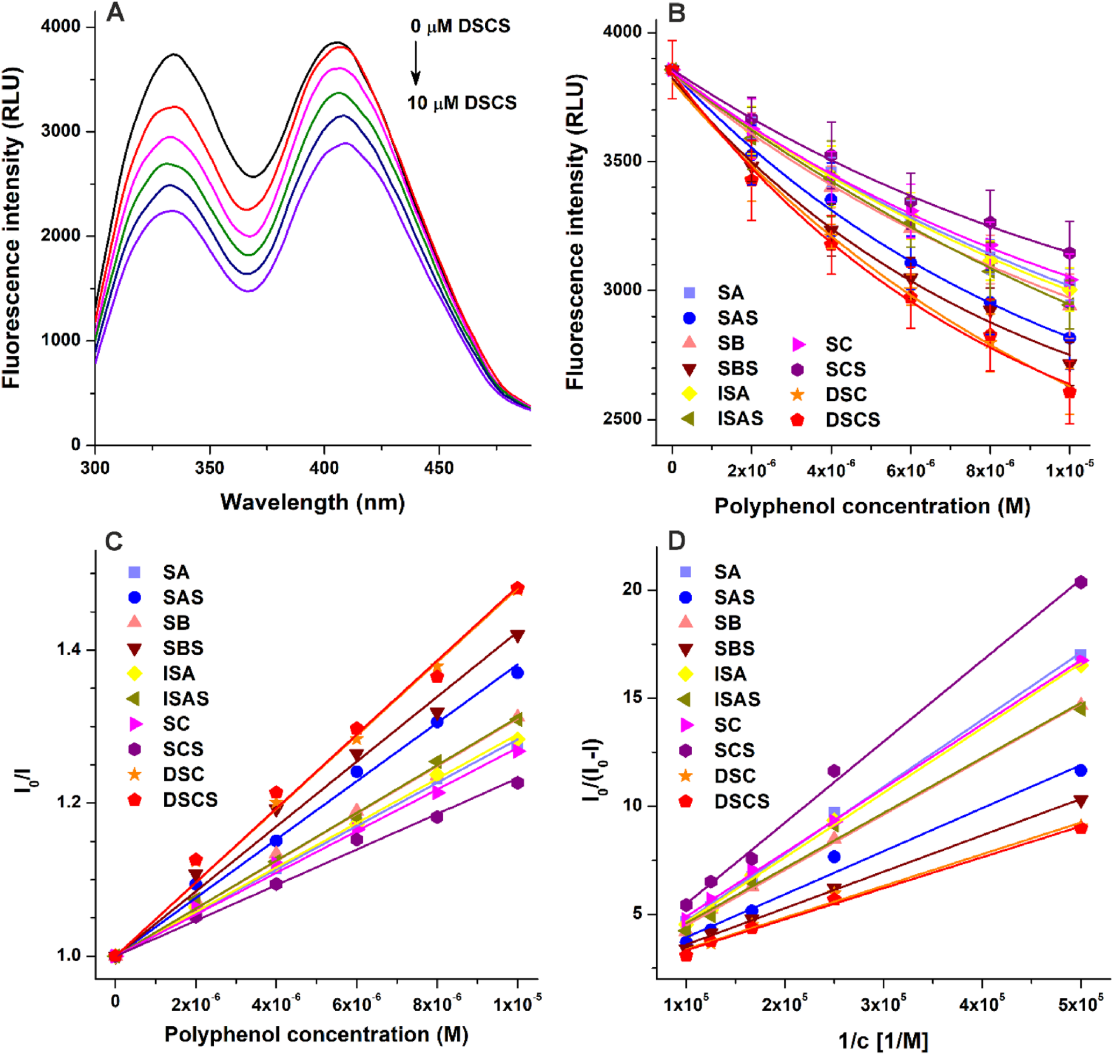
(A) Representative fluorescence emission spectra of XO (0.4 μmol L^−1^) in the presence of increasing concentrations of DSCS (0–10 μmol L^−1^). (B) Polyphenol‐induced decreases in the emission signal of XO, after the correction of inner‐filter effects (*λ*_ex_ = 280 nm, *λ*_em_ = 335 nm). (C) Stern–Volmer plots and (D) modified Stern–Volmer plots of polyphenol–XO complexes (SA, silybin A; SAS, silybin A‐20‐*O*‐sulfate; SB, silybin B; SBS, silybin B‐20‐*O*‐sulfate; ISA, isosilybin A; ISAS, isosilybin A‐20‐*O*‐sulfate; SC, silychristin; SCS, silychristin‐19‐*O*‐sulfate; DSC, 2,3‐dehydrosilychristin; DSCS, 2,3‐dehydrosilychristin‐19‐*O*‐sulfate).

Since we noticed the weak to highly potent inhibitory actions of the different silymarin components/metabolites on XO, we tested the interactions of these polyphenols with the enzyme using fluorescence quenching experiments. Increasing concentrations of polyphenols (0–10 μmol L^−1^) were added to a standard amount of the protein (0.4 μmol L^−1^) and then the emission spectra were recorded (*λ*_ex_ = 280 nm). The inner‐filter effects of polyphenols were corrected based on their absorbance values (see Eqn (1)). Then the changes in the emission signal of XO were evaluated at 335 nm. Under the applied conditions, polyphenols did not exhibit any background fluorescence. Each compound induced concentration‐dependent decreases in the emission signal of XO (Fig. 6). DSCS caused the strongest impact, followed by DSC, SBS, SAS, ISAS, SB, ISA, SA, SC and SCS (Fig. 6(B)). Stern–Volmer plots (Eqn (2); *R* ^2^ = 0.991–0.998; Fig. 6(C)) and modified Stern–Volmer plots (Eqn (3); *R* ^2^ = 0.984–0.999; Fig. 6(D)) showed good linearity. The log *K* values of polyphenol–XO complexes were in the 4.7–5.1 range (Table 2). Based on these data, the enzyme produced the most stable complexes with DSCS, DSC and SBS (log *K* ≈ 5.1); while the weakest interactions were observed with SA, ISA and SCS (log *K* ≈ 4.7).

**Table 2:** Decimal logarithmic values of Stern–Volmer quenching constants (*K*_SV_) and binding constants (*K*) of polyphenol–XO complexes based on fluorescence quenching studies (*λ*_ex_ = 280 nm, *λ*_em_ = 335 nm), where the unit of both *K*_SV_ and *K* is L mol^−1^ (SA, silybin A; SAS, silybin A‐20‐*O*‐sulfate; SB, silybin B; SBS, silybin B‐20‐*O*‐sulfate; ISA, isosilybin A; ISAS, isosilybin A‐20‐*O*‐sulfate; SC, silychristin; SCS, silychristin‐19‐*O*‐sulfate; DSC, 2,3‐dehydrosilychristin; DSCS, 2,3‐dehydrosilychristin‐19‐*O*‐sulfate)

Complex	Log *K*_SV_ ± SEM	Log *K* ± SEM
SA–XO	4.45 ± 0.04	4.71 ± 0.01
SAS–XO	4.58 ± 0.03	4.99 ± 0.05
SB–XO	4.49 ± 0.01	4.87 ± 0.01
SBS–XO	4.63 ± 0.01	5.05 ± 0.02
ISA–XO	4.46 ± 0.01	4.74 ± 0.01
ISAS–XO	4.49 ± 0.01	4.90 ± 0.04
SC–XO	4.43 ± 0.01	4.81 ± 0.01
SCS–XO	4.37 ± 0.03	4.66 ± 0.02
DSC–XO	4.68 ± 0.01	5.11 ± 0.05
DSCS–XO	4.68 ± 0.01	5.12 ± 0.03

### Modeling studies

**Figure 7:**
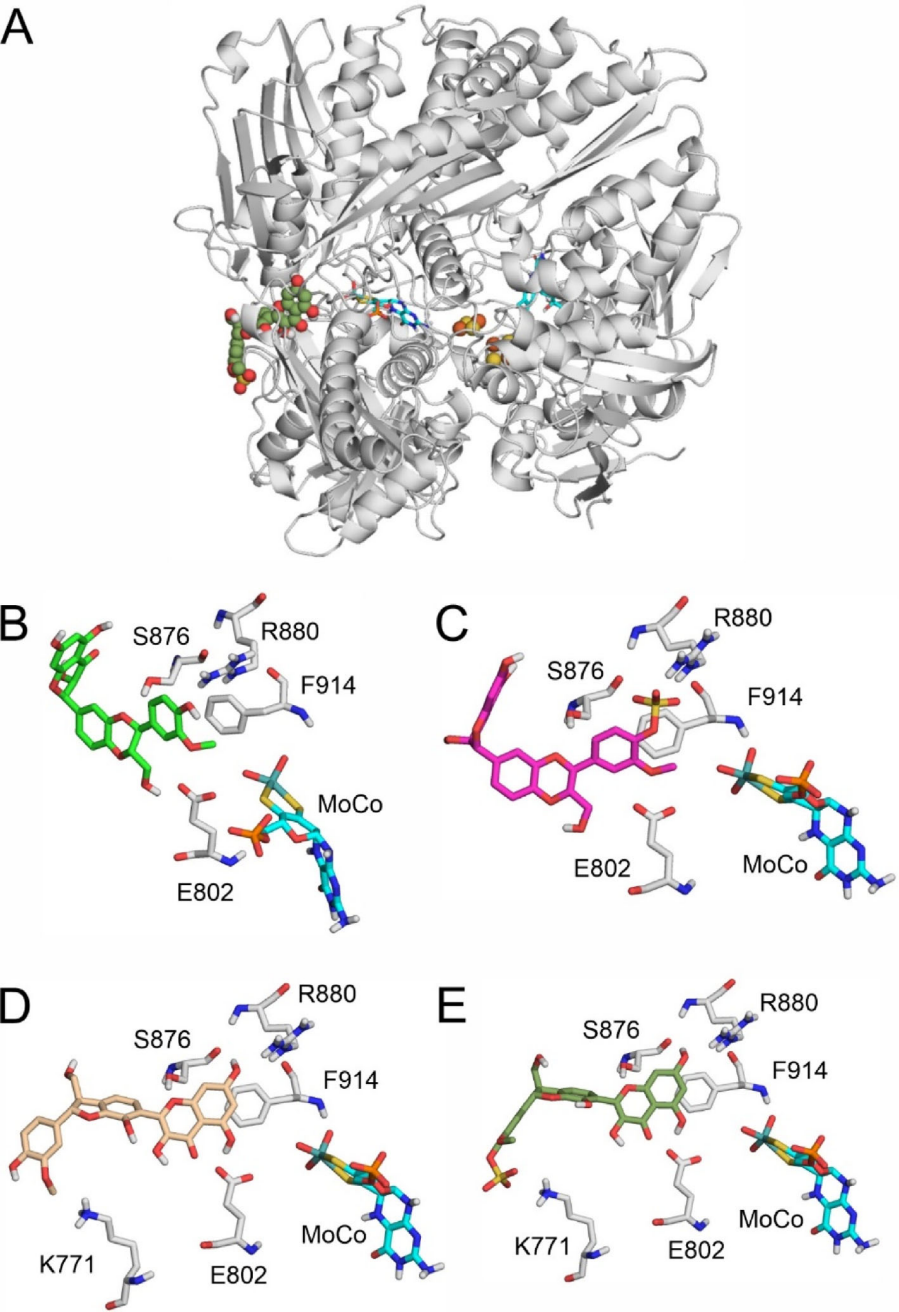
Interaction of DSCS with the active center of XO (A), where XO is represented as grey cartoon, DSCS as green spheres, Fe_2_S_2_ complex as yellow‐orange spheres, and MoCo and FAD as teal sticks. Close‐up images of SB (B), SBS (C), DSC (D) and DSCS (E) binding to the active site of XO (SB, silybin B; SBS, silybin B‐20‐*O*‐sulfate; DSC, 2,3‐dehydrosilychristin; DSCS, 2,3‐dehydrosilychristin‐19‐*O*‐sulfate). Interacting amino acids are highlighted as grey sticks and labeled according to PDB: 3eub.

To find a structural explanation for the experimentally observed XO inhibitory properties, four flavonolignan compounds (SB, SBS, DSC and DSCS) were docked to the active center of XO (Fig. 7).

SB and SBS bound to XO with ring E (according to Valentová *et al*.^38^) pointing towards the MoCo (Fig. 7(B),(C)). The closest heavy atom to MoCo was the carbon atom of the methoxy groups for both SB and SBS. The hydrophilic extensions in ring E (a hydroxyl group for SB and a sulfate group for SBS) pointed towards R880, and ring E was parallel to the phenyl group of F914. E802 and S876 interacted with the hydrophilic extensions of rings B and D (Fig. 7(B),(C)).

DSC and DSCS bound differently to XO (Fig. 7(D),(E)) compared to SB and SBS. Rings A and C of DSC and DSCS (Fig. 1) were pointing towards MoCo, ring E was pointing out of the binding pocket and the positively charged side chain of K771 interacted with the hydrophilic extensions of ring E for both compounds. The closest heavy atom to MoCo was a carbon atom in ring A for both DSC and DSCS, which is similar to the binding mode of xanthine (observed in PDB: 3eub^48^). The hydrophilic extensions of rings A and C interacted with R880, E802 and S876 (Fig. 7(D),(E)).

## DISCUSSION

Hyperuricemia can result in the precipitation of monosodium urate crystals in joints, resulting in the development of gout which is a common inflammatory disease worldwide.^52^ Over the past two decades, the global incidence of gout has increased by 63%, affecting more than 50 million people.^19^ The XO inhibitor APU is the fist‐line urate‐lowering drug used in the pharmacotherapy of gout;^20^ however, due to its side effects, the potential application of phytochemicals is widely studied.^52^ Despite the strong *in vitro* inhibitory effects of certain polyphenols on XO,^25^ there is no strong clinical evidence regarding their suitability in the treatment of gout.^52^ Typically, the high presystemic elimination of polyphenols can strongly limit their *in vivo* antihyperuricemic effects.^26^ Therefore, the improvement of the oral bioavailability of polyphenols as well as the impacts of their metabolites on XO may have high pharmacological importance. Polyphenols, including catechins, stilbenoids and flavonolignans, are abundant in nature and are also contained by certain dietary supplements and/or medications. In the current study, we examined the inhibitory effects of catechins, RES, silymarin flavonolignans and some of their metabolites on XO.

In agreement with earlier reports,^29^, ^30^, ^31^ we also noticed weak inhibitory actions of tea catechins on XO, where gallate esters (ECG (Fig. 1) and EGCG) showed stronger impacts compared to CAT, ECT and EGC (Fig. 2). Furthermore, similar to other studies,^32^, ^33^ a weak RES‐induced inhibition of XO was observed (Fig. 2). Glucuronidation considerably decreased while sulfate conjugation did not affect the inhibitory action of RES (Fig. 2). These results are in accordance with our previous observations that glucuronides are typically weaker while sulfate derivatives are usually similar or even stronger inhibitors of XO compared to the parent polyphenols.^27^, ^28^ Based on these data, it is unlikely that catechins and RES could affect uric acid levels *in vivo*.

Previous studies suggested the weak inhibitory action of silybin (diastereomeric mixture of SA and SB) on XO.^34^, ^35^ Our data showed the same: SA and SB were the least potent inhibitors of XO among the silymarin components/metabolites examined (Fig. 2). ISA was also a relatively weak inhibitor (Fig. 2), while SC was a moderate inhibitor of the enzyme (Table 1). Unexpectedly, DSC proved to be a highly potent inhibitor of xanthine oxidation, showing significant inhibitory effects even at low nanomolar concentrations (Fig. 2) and exhibiting approximately threefold stronger impact than the positive control APU (Table 1). These findings suggest that, regarding rings B and D, the dihydrobenzofuran structure (in SC and DSC; see Fig. 1 and supporting information, Fig. S1) is more favorable compared to the benzodioxan moiety (in SA, SB and ISA; see supporting information, Fig. S1), and markedly increases the inhibitory effect on XO. Furthermore, DSC proved to be a 50‐fold stronger inhibitor of xanthine oxidation compared to SC (Table 1), which strongly underlines the importance of the 2–3 double bond in ring C (Fig. 1).

The sulfate conjugation of SA, SB and ISA considerably increased their inhibitory effects on xanthine oxidation, resulting in nanomolar (SBS and ISAS) or low micromolar (SAS) IC_50_ values (Table 1). Furthermore, DSCS proved to be a slightly stronger inhibitor compared to DSC. In accordance with these observations, the sulfate derivatives of flavonoids quercetin, luteolin, myricetin, naringenin and ampelopsin exerted more potent inhibitory effects on XO than the parent aglycons.^27^, ^28^ However, SCS was a weaker inhibitor of xanthine oxidation compared to SC (Fig. 2), showing that sulfate conjugation can sometimes decrease the inhibitory potency, as we also noticed regarding the flavonoid chrysin.^53^

No data are available about the cellular or plasma concentrations of the sulfate derivatives of silymarin components. However, in a recent study, the metabolic profile of silymarin constituents was analyzed in urine and feces of healthy human volunteers.^16^ The levels of sulfate derivatives in urine showed major interindividual variations: after 10 days treatment with silymarin (200 mg, twice daily), the semiquantitative percentage of sulfates was between 0% and 58%; while it was in the 0–19% range after 90 days. Therefore, it is very difficult to provide a good prediction regarding the *in vivo* antihyperuricemic effects of the sulfate metabolites formed. Another important observation is the highly potent inhibitory action of DSC on XO. In a previous study, a detailed analysis of silymarin components was performed for six various silymarin preparations, where the DSC contents were observed in the range 0.0–1.5%.^54^ Considering the very low amount of DSC in silymarin, further *in vivo* studies are reasonable to explore its potential antihyperuricemic impact, because it seems to be a novel potential candidate in the treatment of gout and the corresponding higher cardiovascular risk. Nevertheless, as possible limitations, the low oral bioavailability and the relatively fast elimination of these compounds should also be considered.

Another interesting issue is the inhibitory actions of flavonolignans on XO‐catalyzed 6MP oxidation. Catechins, RES, R3S and R3G had similar effects with both substrates (Figs 2 and 3), they being weak inhibitors of 6MP oxidation. Therefore, the elimination of the drug is likely not influenced even by a high consumption of these polyphenols. Most of the silymarin components/metabolites were somewhat stronger inhibitors of xanthine *versus* 6MP oxidation. Based on the IC_50_ values determined, SAS, SBS, ISAS, SC, DSC and DSCS are two‐ to fourfold weaker inhibitors of 6MP oxidation, with the largest difference shown by ISAS (Table 1). Nevertheless, SBS, DSC and DSCS also presented nanomolar IC_50_ values in the 6MP assay. As has been reported, APU is a considerably weaker inhibitor of 6MP oxidation;^27^, ^28^ therefore, SAS, SBS, ISAS, DSC and DSCS exerted stronger inhibitory actions on the XO‐catalyzed 6MP oxidation compared to the positive control inhibitor (Table 1). As we discussed above, the tissue and plasma levels of these sulfate metabolites are unknown. Considering that the typical total peak plasma concentrations of silymarin (components and metabolites together) are in the nanomolar to the low micromolar range^17^, ^18^, ^55^ and considering the low amounts of DSC in silymarin,^54^ it seems to be unlikely that silymarin treatment could interfere with the XO‐mediated elimination of 6MP under clinical conditions.

The binding constants of flavonolignan–XO complexes were examined based on fluorescence quenching studies. The log *K* values (Table 2) were mostly in agreement with the inhibitory potency (Table 1), thus a higher binding constant typically accompanied a stronger inhibitory effect on XO. The *K* values regarding the complexes of the three strongest inhibitors (SBS, DSC and DSCS) were above 10^5^ L mol^−1^. Furthermore, the least stable complexes were SA–XO, ISA–XO and SCS–XO (log *K* ≈ 4.7). Interestingly, SB showed a slightly higher binding constant (log *K* ≈ 4.9) despite its weak inhibitory action (Fig. 1). In addition, we found less than threefold difference between the *K* values of the strongest *versus* the weakest complexes, suggesting that the binding position of silymarin components/metabolites on XO may have high importance besides the binding affinity.

Unfortunately, the results of modeling studies did not explain the more potent inhibitory action of SBS compared to SB. This may be caused by the stronger interaction of SBS with the enzyme (Table 2) and/or by the bulkier structure of the sulfate moiety which occupies a relatively close binding position to MoCo (Fig. 7(C)). However, in agreement with the experimental results (Table 1), modeling studies suggested that DSCS binds to XO with higher affinity than DSC, resulting from the ionic interaction of K771 with its sulfate group (Fig. 7(E)). Furthermore, the similar binding mode of DSC and DSCS compared to xanthine may explain their outstanding inhibitory potency.

This explorative study demonstrates the reversible, highly potent inhibitory effects of SBS, DSC and DSCS on XO. As an important limitation, our *in vitro* results cannot be extrapolated to clinical data, because we do not have enough information regarding the pharmacokinetic properties of DSC, and no data are available concerning the plasma and tissue levels of SBS, DSC and DSCS. Therefore, an investigation of the *in vivo* pharmacokinetics and antihyperuricemic impacts of DSC should be conducted in the future.

## CONCLUSIONS

In summary, the effects of tea catechins, RES, silymarin flavonolignans and some of their conjugated metabolites were examined on XO‐catalyzed xanthine and 6MP oxidation. Catechins, RES, R3S and R3G showed no or only weak inhibitory effects on XO. SA, SB and ISA were weak inhibitors of the enzyme, while their potency was strongly increased by sulfate conjugation. SC exerted moderate inhibitory action, and its effect was decreased by sulfate substitution. Since both DSC and its sulfate conjugate (DSCS) proved to be highly potent inhibitors of XO, DSC seems to be a promising candidate to test its *in vivo* antihyperuricemic effects.

## FUNDING INFORMATION

The work of MP is supported by the Hungarian National Research, Development and Innovation Office (FK138184) and by the János Bolyai Research Scholarship of the Hungarian Academy of Sciences (BO/00381/21). KV was supported by the Czech Science Foundation (23‐04654S). This paper was supported by the János Bolyai Research Scholarship of the Hungarian Academy of Sciences. The work of CH was supported by the Hungarian National Research, Development and Innovation Office (PharmaLab, RRF‐2.3.1‐21‐2022‐00015).

## CONFLICT OF INTEREST

The authors declare no competing interests.

## AUTHOR CONTRIBUTIONS

**Tímea Bencsik:** Formal analysis, Investigation; **Orsolya Balázs:** Investigation; **Róbert G Vida:** Investigation; **Balázs Z Zsidó:** Formal analysis, Investigation; **Csaba Hetényi:** Formal analysis, Funding acquisition, Investigation, Methodology, Validation, Writing; **Kateřina Valentová:** Funding acquisition, Investigation, Methodology, Resources, Validation; **Miklós Poór:** Conceptualization, Formal analysis, Funding acquisition, Investigation, Methodology, Supervision, Validation, Writing.

## Supporting information


**Data S1.** Supporting Information.

## Data Availability

The data that support the findings of this study are available from the corresponding author upon reasonable request.
