# Supplementary material for: Effects of catechins, resveratrol, silymarin components and some of their conjugates on xanthine oxidase‐catalyzed xanthine and 6‐mercaptopurine oxidation
Source: J Sci Food Agric. 2024 Nov 28;105(5):2765–76. doi: 10.1002/jsfa.14045 (PMC11909324; doi:10.1002/jsfa.14045)
Supplement: Supplementary file 1 — Data S1. Supporting Information. [file JSFA-105-2765-s001.docx]

**Effects of catechins, resveratrol, silymarin components, and some of their conjugates on xanthine oxidase-catalyzed xanthine and 6-mercaptopurine oxidation**

SUPPLEMENTARY MATERIAL

Tímea Bencsik ^1^, Orsolya Balázs ^2^, Róbert G. Vida ^2^, Balázs Z. Zsidó ^3,4^, Csaba Hetényi ^3,4^, Kateřina Valentová ^5^, Miklós Poór ^6,7,*^

^1^ Department of Pharmacognosy, Faculty of Pharmacy, University of Pécs, Rókus u. 4, H-7624 Pécs, Hungary

^2^ Department of Pharmaceutics and Central Clinical Pharmacy, Faculty of Pharmacy, University of Pécs, Rókus u. 2, H-7624 Pécs, Hungary

^3^ Pharmacoinformatics Unit, Department of Pharmacology and Pharmacotherapy, Medical School, University of Pécs, Szigeti út 12, H-7624 Pécs, Hungary

^4^ National Laboratory for Drug Research and Development, H-1117 Budapest, Hungary

^5^ Institute of Microbiology of the Czech Academy of Sciences, Vídeňská 1083, CZ-142 00 Prague, Czech Republic

^6^ Department of Laboratory Medicine, Medical School, University of Pécs, Ifjúság útja 13, H-7624 Pécs, Hungary

^7^ Molecular Medicine Research Group, János Szentágothai Research Centre, University of Pécs, Ifjúság útja 20, H-7624 Pécs, Hungary

*Corresponding author: Miklós Poór, PharmD, PhD

Department of Laboratory Medicine,

Medical School, University of Pécs

Ifjúság útja 13, H-7624 Pécs, Hungary

Phone: +36-72-501-500 ext: 29250

E-mail: [poor.miklos@pte.hu](mailto:poor.miklos@pte.hu)


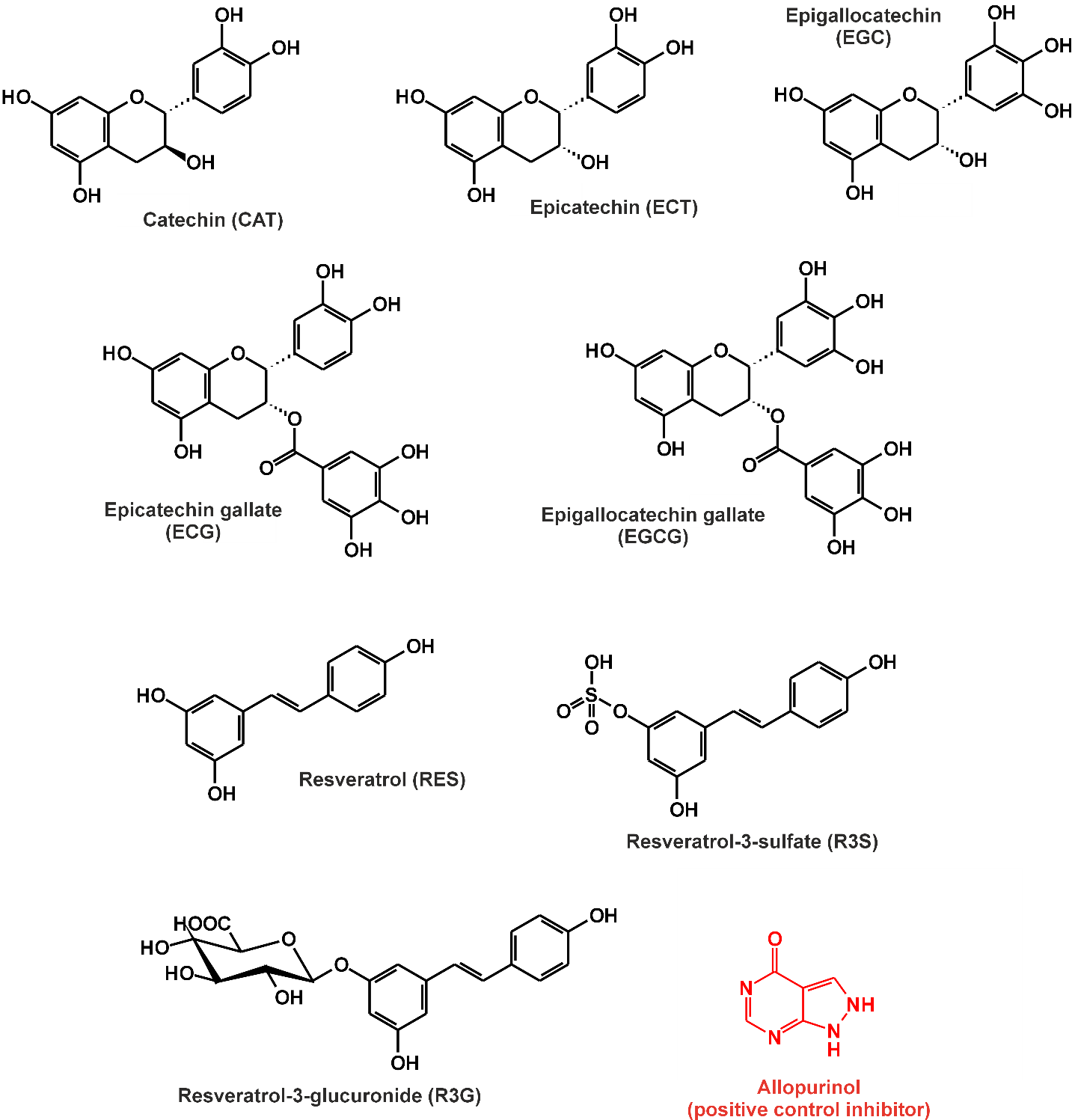


*Continued on the next page.*


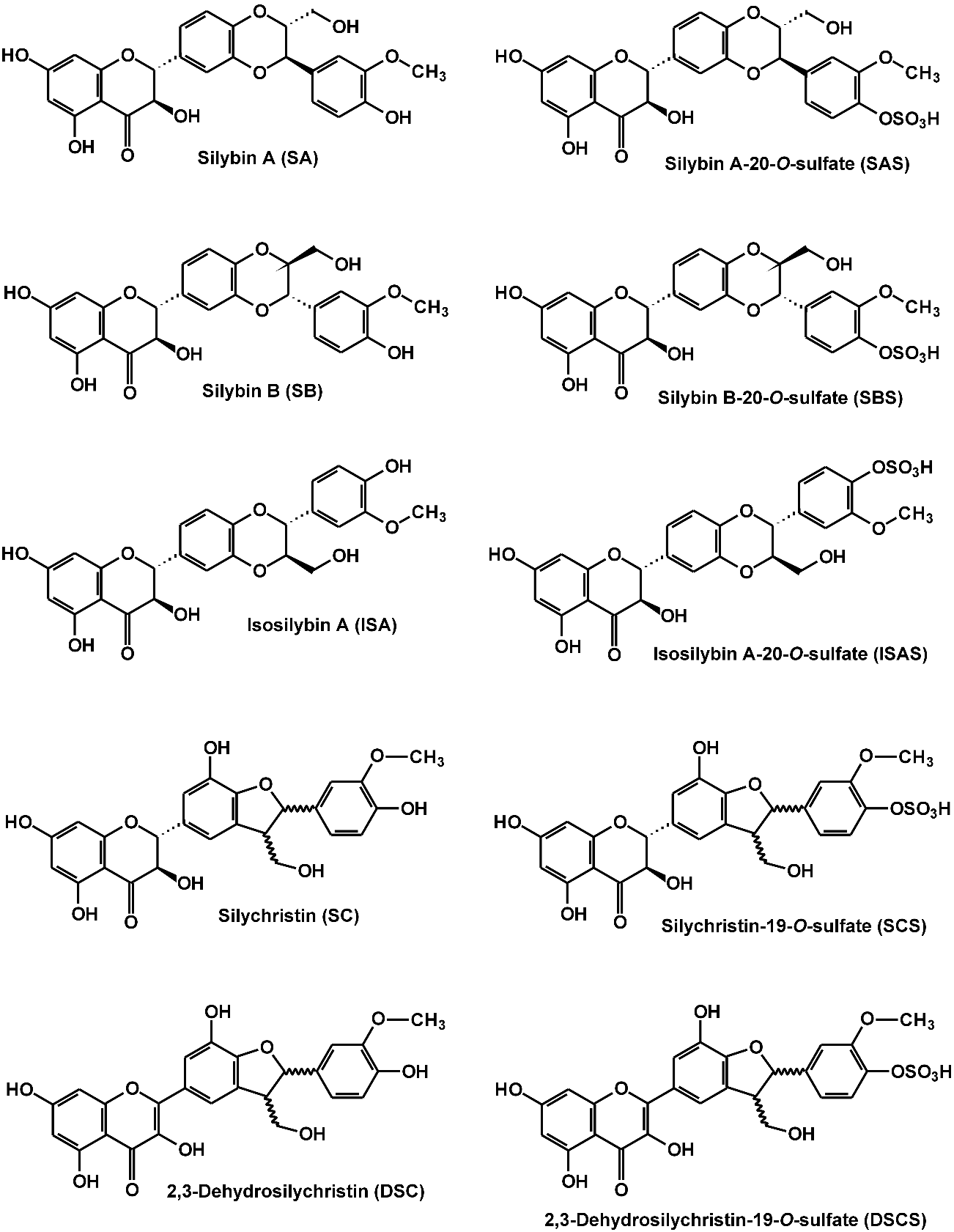


**Fig. S1**: Chemicals structures of the polyphenols examined (catechins, resveratrol and its conjugates, and silymarin components and their sulfate derivatives) and allopurinol (positive control inhibitor, marked with red color).

**
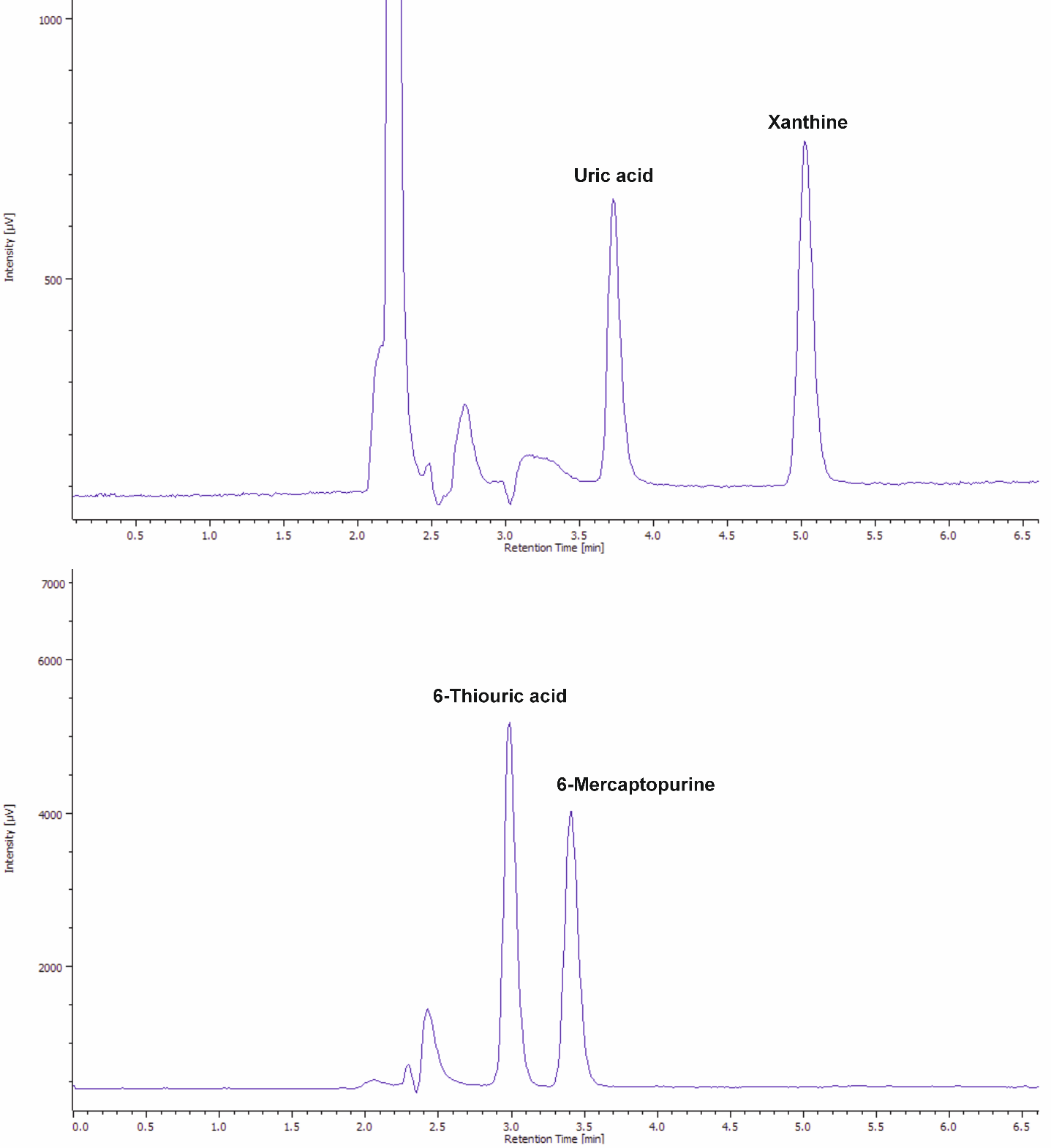
**

**Fig. S2**: Representative chromatograms regarding xanthine–uric acid (top; each 1 μM) and 6-mercaptopurine–6-thiouric acid (bottom; each 1 μM) HPLC assays.
